# Nature of Self-Trapped Exciton Emission in Zero-Dimensional Cs_2_ZrCl_6_ Perovskite Nanocrystals

**DOI:** 10.1021/acs.jpclett.3c01878

**Published:** 2023-08-21

**Authors:** Yanmei He, Siping Liu, Zehan Yao, Qian Zhao, Pavel Chabera, Kaibo Zheng, Bin Yang, Tönu Pullerits, Junsheng Chen

**Affiliations:** † Department of Chemical Physics and NanoLund, Lund University, P.O. Box 124, 22100 Lund, Sweden; ‡ Nano-Science Center & Department of Chemistry, University of Copenhagen, Universitetsparken 5, 2100 Copenhagen, Denmark; § State Key Laboratory of Molecular Reaction Dynamics, Dalian Institute of Chemical Physics, Chinese Academy of Science, 116023 Dalian, P. R. China; ∥ Guangxi Key Laboratory of Chemistry and Engineering of Forest Products, School of Chemistry and Chemical Engineering, Guangxi Minzu University, Nanning 530006, P. R. China; ⊥ Department of Chemistry, Technical University of Denmark, DK-2800 Kongens Lyngby, Denmark

## Abstract

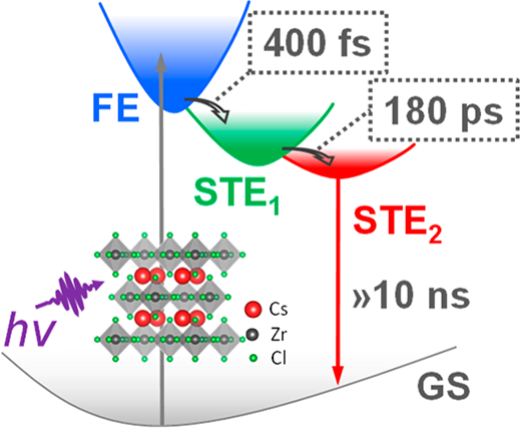

Low dimensional perovskite-inspired materials with self-tapped exciton (STE) emission have stimulated a surge of cutting-edge research in optoelectronics. Despite numerous efforts on developing versatile low-dimensional perovskite-inspired materials with efficient STE emissions, there is little emphasis on the intrinsic dynamics of STE-based broad emission in these materials. Here, we investigated the excited state dynamics in zero-dimensional (0D) Cs_2_ZrCl_6_ nanocrystals (NCs) with efficient blue STE emission. By using femtosecond transient absorption (fs-TA) spectroscopy, the ultrafast STE formation process within 400 fs is directly observed. Then, the formed STEs relax to an intermediate STE state with a lifetime of ∼180 ps before reaching the emissive STE state with a lifetime of ∼15 μs. Our work offers a comprehensive and precise dynamic picture of STE emission in low-dimensional metal halides and sheds light on extending their potential applications.

Halide perovskites have garnered significant attention and extensive research as compelling candidates for optoelectronic applications. It is primarily due to their remarkable properties, including a high absorption coefficient, impressive luminescence efficiency, and long carrier diffusion lengths.^1−11^ These optical and electrical properties can be effectively tuned by changing their chemical composition.^12−17^ As the chemical composition directly influences the dimensionality of perovskites, low-dimensional structures are easy to obtain by altering metal cations, mixing halide ions, and using long chain organic ligands. In low dimensional metal-halides, the strong lattice distortion induced by the pseudo-Jahn–Teller effect can enhance the electron–phonon interactions.^18−21^ Furthermore, since the charge carriers (excitons) are well confined, the exciton binding energy is large, leading to an efficient exciton radiative recombination and high photoluminescence quantum yield (PLQY). These unique characteristics of low-dimensional halide perovskites make them attractive for light-emitting diode (LED) applications.^22−24^ Especially, they can exhibit broadband emission with a large Stokes shift, rendering the possibility to achieve direct single-component white-light LEDs.^25−27^

The origin of such a broadband emission in low-dimensional halide perovskites has been assigned to different photophysical processes, such as self-trapped excitons (STEs),^8,18,19,28,29^ in-gap states caused by defects,^30^ momentarily trapped exciton polarons,^31^ and the coupling of triplet states in inorganic as well as organic barrier layers.^32^ The low dimensional halide perovskites are often featured by soft and ionic lattices, where the inorganic or organic cations interact with the anionic octahedra via ionic interactions. Upon photoexcitation, lattice distortion could happen, and then, the electrons or holes are easily localized and strongly interact with phonons to form so-called STEs/polarons. Compared to the mentioned extrinsic trap state or permanent defect in the lattice, STEs will disappear once the photoexcitation is removed. When the STEs recombine radiatively, they may show an extremely high PLQY; for example, in 2018, Hosono et al. first reported zero-dimensional (0D) Cs_3_Cu_2_I_5_ single crystals with a PLQY of ∼90%.^33^ Inspired by this successful attempt, different copper-based perovskite-inspired materials with high PLQY were developed.^34^ Recently, many double perovskites with high PLQY are also reported by doping different metal ions (e.g., Bi^3+^, Te^4+^, and Sb^3+^).^24,35−39^

Numerous efforts have been devoted to developing novel low dimensional perovskite-inspired materials with efficient STE emissions. However, the more fundamental studies addressing the photophysics and dynamics of STEs in low dimensional perovskite-inspired materials are still rare.^40,41^ A thorough understanding of the formation and relaxation processes of STEs is significant to further improve their performance and develop new low dimensional perovskite-inspired materials for advanced optoelectronic applications. The recently reported 0D vacancy-ordered double perovskite Cs_2_ZrCl_6_ showed typical STE emission with high efficiency and outstanding chemical stability, ensuring its potentiality in versatile applications including LEDs and X-ray scintillator screens.^8,42^ Herein, we explored the photoinduced exciton dynamics of these Cs_2_ZrCl_6_ nanocrystals (NCs) by utilizing ultrafast time-resolved optical spectroscopies, which can obtain a deep understanding of the STEs in both Cs_2_ZrCl_6_ and its perovskite counterparts. The as-synthesized NCs are of high quality and exhibit broad blue STE emission. The formation and relaxation process of STEs and the related broadband PL are discussed comprehensively.

The Cs_2_ZrCl_6_ NCs were synthesized by following a reported hot-injecting method (see details in the Supporting Information).^8^ As shown in Figure 1a (inset), the prepared Cs_2_ZrCl_6_ NCs with blue emission were homogeneously dispersed in heptane, exhibiting a high PLQY of ∼50%. Confirmed by the transmission electron microscopy (TEM) images, the Cs_2_ZrCl_6_ NCs exhibit a quasi-spherical shape with an average crystal size of 17 ± 2 nm (Figure 1b and c). By resolving the crystal structure from the powder X-ray diffraction (PXRD) refinement, we found the diffraction peaks of NCs match very well with the standard pattern of Cs_2_ZrCl_6_ (Figure 1e, PDF#74-1001, cubic space group *Fm*3*m* a = b = c = 10.407 Å, α = β = γ = 90°), which is the typical vacancy-ordered double perovskite 0D structure (Figure 1f). Figure 1a shows the absorption, excitation, and emission spectra of Cs_2_ZrCl_6_ NCs dispersed in heptane. The absorption spectrum features a strong excitonic absorption peak at 250 nm, corresponding to the lowest excited state transition. The PL excitation (PLE) spectrum matches with the absorption peak at 250 nm. A weak absorption at the range from 280 to 400 nm is due to the absorption of residual oleylamine and oleic acid in the NC solution (Figure S1). All the observations in the absorption spectrum, TEM, and PXRD are in line with our and other groups’ previous reports.^8,43−45^ Under 250 nm excitation, the Cs_2_ZrCl_6_ NCs show a broad PL emission centered at 450 nm (Stokes shift of ∼195 nm), a large full width at half-maximum (FWHM) of 129 nm, and a long PL lifetime (∼15 μs, Figure 1d). These spectral characteristics reveal the typical STE behavior of low-dimensional metal perovskite-inspired materials.

**Figure 1:**
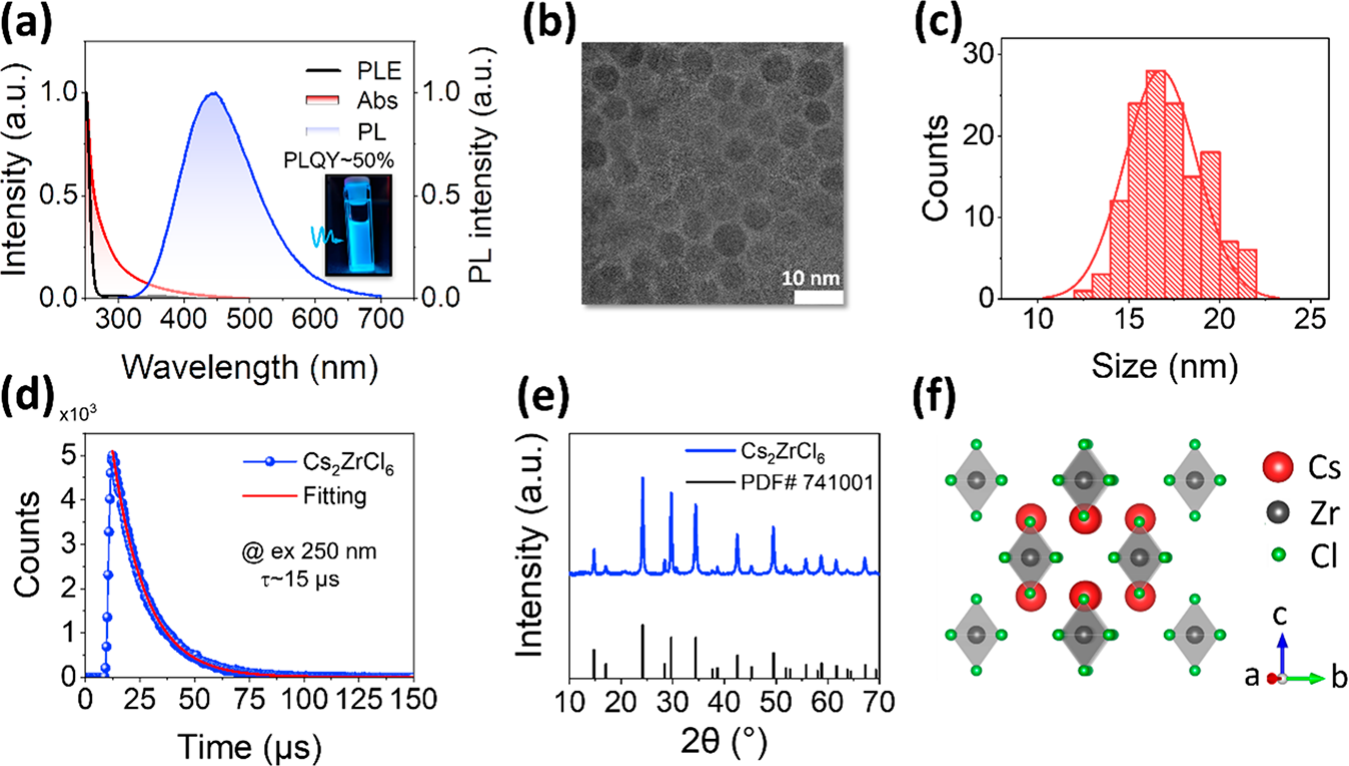
(a) UV–vis absorption, PL and PLE spectra of Cs_2_ZrCl_6_ NCs (Inset: Cs_2_ZrCl_6_ NCs under 254 nm UV light excitation showing the PLQY of ∼50%). (b) TEM image of Cs_2_ZrCl_6_ NCs (scale bar: 10 nm). (c) Size distribution histogram extracted from TEM images. (d) Time-resolved PL spectrum of Cs_2_ZrCl_6_ NCs excited at 250 nm with fitting results. (e) PXRD pattern of Cs_2_ZrCl_6_ NCs measured at room temperature. The gray line stands for the standard PXRD pattern. (f) Crystal structure of the Cs_2_ZrCl_6_ NCs.

The microsecond time scale dynamics of STEs in Cs_2_ZrCl_6_ NCs can be observed from the time-resolved PL based on time-correlated single-photon counting (TCSPC) measurements (Figure 1d), which agrees well with earlier studies.^8,21^ So far, little is known about the formation of STEs and their relaxation processes at subpicosecond to picosecond time scales in Cs_2_ZrCl_6_ NCs.^40,41,46^ In our analysis, we employed the streak camera technique, which offers a time resolution of tens of picoseconds, to examine the radiative recombination process of STEs. This method is complementary to the TCSPC technique. Furthermore, for studying the formation and nonradiative relaxation processes of STEs, we utilized ultrafast femtosecond transient absorption (fs-TA) spectroscopy, which enables the time resolution down to the subpicosecond scale.

In Figure 2, we show the time-resolved PL spectra of NCs measured by a streak camera under the excitation of 266 nm with femtosecond laser pulses. The time-resolved PL emission shows a spectral shift (from blue to red) with time (Figure 2a and b), while we cannot resolve two distinct PL peaks due to the spectral overlap and the limited signal-to-noise ratio, attributed to the long PL lifetime (microsecond time scale, Figure 1d) and the high repetition rate (80 MHz) of the laser pulses in our streak camera measurement. Furthermore, in this condition, there might be an accumulation effect which influences the PL decay kinetics. To determine if it is the case for the current system, we measured the PL decays at different excitation fluences. The PL decay kinetics are nearly the same at different excitation fluences ranging from 5.6 × 10^12^ to 1.4 × 10^14^ photons/cm^2^/pulse (Figure 2c). We could conclude (at least in this detection time-window) the accumulation effect does not play a significant role for the PL decay kinetics. To gain a quantitative result for the PL decays, we used the global fitting method to analyze the time-resolved PL spectra. Here, a sum-of-exponential-decay model was used to fit the data. In multiexponential decay dynamics, the spectra of decay components greatly facilitate analysis. As shown in Figure 2d, in the absence of further external information, we got these spectra from the minimally complex representation of multiexponential decays: the so-called decay associated spectra (DAS) and evolution associated spectra (EAS). In the DAS model, the components are noninteracting and decay in parallel. Conversely, the EAS approach imposes a sequential and unidirectional relaxation model. In this scenario, the spectra can well reveal involved excited states in the whole relaxation process because of a good approximation to the actual state–state processes. After global fitting by using these two models, we obtained two components: a short lifetime of around 200 ps and a lifetime much longer than the detection time-window (≫ 2 ns). The long lifetime component can be assigned to the PL emission observed from the steady-state PL spectrum, as they show a very similar spectral shape with an emission peak located at 450 nm. The short lifetime component (200 ps) indicates there might be an intermediate excited state between the initial excited state and the final emissive STE state (with a lifetime of ∼15 μs).

**Figure 2:**
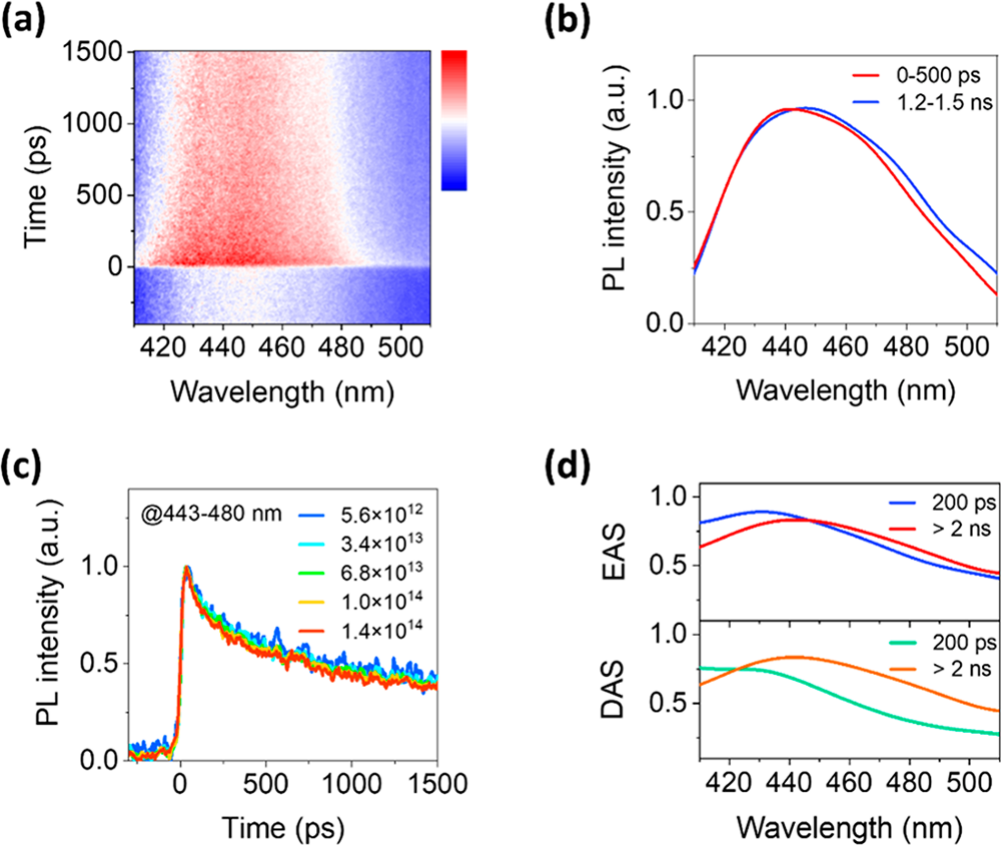
(a) Time-resolved PL spectra and (b) spectral evolution of Cs_2_ZrCl_6_ NCs measured by a streak camera (excited at 266 nm). (c) Normalized streak camera based-TRPL decay traces of Cs_2_ZrCl_6_ NCs at different excitation fluences ranging from 5.6 × 10^12^ to 1.4 × 10^14^ photon/cm^2^/pulse. (d) Comparison of DAS and EAS components derived from singular value decomposition (SVD) global fitting.

Based on previous reports, the formation time of STEs is in the subpicosecond or few picoseconds time scale (e.g., ∼500 fs) in low-dimensional perovskite-inspired materials,^47^ while our streak camera approach with an instrument response function (IRF ∼ 20 ps) is not sufficient to observe the formation of STEs. Therefore, we turned to ultrafast femtosecond transient absorption spectroscopy (fs-TA) to study the excited state dynamics of STEs in Cs_2_ZrCl_6_ NCs. The samples were excited at 266 nm (*hυ* = 4.66 eV) whose energy is nearly the same as (or close to) the band-edge excitation energy (E_g_ = 4.70 eV). For the probe, we used a supercontinuum white light covering the entire visible light region.

Figure 3a shows the fs-TA spectra of the Cs_2_ZrCl_6_ NCs. Upon laser pulse excitation, a very broad positive signal is formed with three peaks located at 440, 540, and 640 nm, respectively. The absence of ground state bleaching (GSB) and stimulated emission (SE) signals within our detection wavelength window is attributed to the ultraviolet absorption (out of detection window) and weakly allowed optical transition dipole moment of STE emission. The broadband excited state absorption (ESA) signal has been assigned to a characteristic feature of STEs in perovskite-inspired materials.^21^ Rather rich spectral dynamics within the first 1 ps are shown in fs-TA spectra: three ESA peaks presented a quick build-up process (<500 fs, Figures 3b and 4a) and then followed with red shifts (shifting to 470, 548, and 650 nm for the peak centered at 440 nm, 540, and 640 nm, respectively). The early time scale spectral dynamics reveal that the STEs in Cs_2_ZrCl_6_ NCs are instantaneously formed in a rather fast time scale. At the time range from 1 to 100 ps, we observed that the ESA signal decays without clear spectral shifts or changes. It cannot be related to the radiative recombination process of STEs in Cs_2_ZrCl_6_ NCs at a microsecond (Figure 1d) time scale.^8^ Therefore, it might be assigned to the relaxation process of an intermediate excited state, which was observed in the time-resolved PL spectra. From 100 ps to 4 ns, the ESA signal featuring three main peaks continues to decay with a much slower rate compared to that from 1 to 100 ps. Furthermore, the spectrum evolves to a featureless broadband from 500 to 750 nm. Interestingly, a new peak started to appear on the blue side of the detection window. Due to the limited spectral and time detection window, we cannot resolve the new peak completely. However, based on the long PL lifetime of STEs, we can assign the featureless broadband ESA signal and new peak to the symbolization of the emissive STE appearance in Cs_2_ZrCl_6_ NCs.

**Figure 3:**
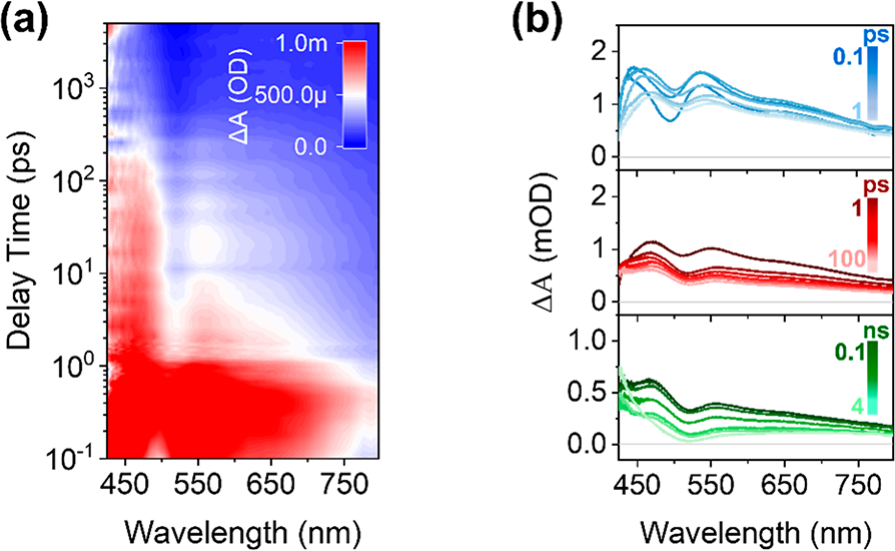
(a) Pseudocolor representation fs-TA spectra of Cs_2_ZrCl_6_ NCs excited at 266 nm with the excitation intensity of 8.3 × 10^13^ photon/cm^2^/pulse (corresponding to ⟨*N*⟩ ∼ 0.3). (b) Transient spectra extracted from panel (a) over several time ranges of interest. Spectra shown at probe delays of the following: top panel: 0.1 (deep blue), 0.25, 0.38, 0.55, 0.70, 0.85, 1 ps (light blue); middle panel: 1 (red), 2, 5, 10, 20, 50, 100 ps (pink); bottom panel: 0.1 (green), 0.15, 0.32, 0.63, 1, 4 ns (light green).

**Figure 4:**
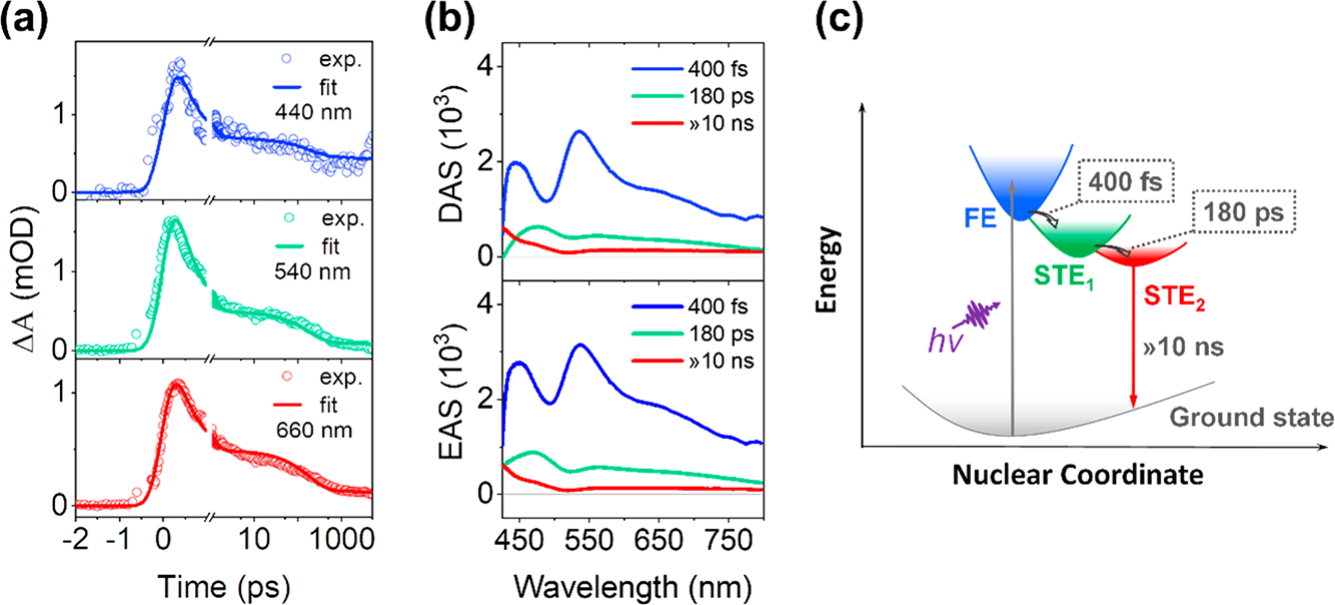
Cs_2_ZrCl_6_ NCs are excited at 266 nm under the excitation fluence of 8.3 × 10^13^ photon/cm^2^/pulse (corresponding to ⟨*N*⟩ ∼ 0.3). (a) Temporal kinetics at 440, 540, and 660 nm for comparison. (b) DAS and EAS components derived from SVD global fitting. (c) Schematic mechanism illustrating the self-trapped excited state dynamics in Cs_2_ZrCl_6_ NCs.

To get a deeper understanding of the comprehensive dynamic picture of STEs, the SVD global fitting is used to quantitatively analyze their formation and relaxation processes. The DAS and EAS extracted from global fitting results are shown in Figure 4b.^48^ From the fs-TA spectra, we found that the initial relaxation from the Franck–Condon state proceeds on a 100 fs time scale based on the rising of the ESA signal (Figure 3b). The process is rather fast, and we do not try to resolve the time scale in our global fitting analysis. Furthermore, considering the excitation energy is close to the bandgap, we do not expect any clear hot-electron cooling process. Therefore, the STE formation process directly observed in the fs-TA spectra was further analyzed by the DAS and EAS results. As shown in Figure 4b, the STE formation process happens on the time scale of 400 fs, which appears to be accompanied by changes of amplitude differences between the two peaks (440 and 540 nm). Overall, the spectral dynamics suggest that substantial changes in electronic structures took place over this notably short time scale.

After completion of the STE formation process, two decay components contributed to the spectral dynamics: First, the two distinct ESA peaks (440 and 540 nm) become blurry with a time scale of ∼180 ps, which is in line with the alterations of the broad ESA signal outlined in the qualitative analysis (spectral evolution shown in Figure 3b). Then, it is followed by further broadening of the ESA peak and appearance of a new ESA peak at the blue edge of the spectral detection window. However, this component decays for such a long time that it is out of the experimental time detection window. Considering Cs_2_ZrCl_6_ NCs exhibit a long PL lifetime of over tens of microseconds (Figure 1d), the long-lived component could be assigned to the final emissive STE state. Such an assignment is not unambiguous, as our experimental time-window is orders of magnitude shorter than the emissive STE state.

The component with a lifetime of 180 ps matches well with the time-resolved PL based on streak camera measurements. Based on its spectral feature, we can assign this component to an intermediate excited state. This intermediate excited state can have a different nature, such as a shallow local minimum configuration of the photoexcited state or Auger recombination process. We carried out excitation fluence dependent PL measurements by using the same pump laser pulse (Figure S2). The integrated PL intensity shows a linearity increase with the excitation fluence before reaching the threshold (0.31 mJ·cm^–2^, corresponding to 8.3 × 10^14^ photon/cm^2^/pulse). In our TA experiment, the excitation fluence (8.3 × 10^13^ photon/cm^2^/pulse) is 1 order of magnitude below the threshold. Furthermore, we estimated the absorption cross-section of the Cs_2_ZrCl_6_ NCs based on inductively coupled plasma optical emission spectroscopy (ICP-OES) (details in the Supporting Information). The calculated average photoexcited exciton number (⟨*N*⟩) per NC is 0.3 under the excitation fluence of 8.3 × 10^13^ photon/cm^2^/pulse. At such excitation fluence, the possibility for generating multiple excitons in one NC is rather low based on the Poisson distribution. Hence, we can rule out the Auger process for the 180 ps component. Based on our previous report, the thermally activated delayed fluorescence (TADF) was observed in Cs_2_ZrCl_6_ NCs, in which the photoluminescence shows a temperature-dependence characteristic: PL intensity is proportionally increasing with increasing the temperature. Until 260 K, the PL intensity decreases when the temperature is further increased.^8^ However, based on the current results, we still cannot rule out other possibilities. We named it an intermediate STE state.

Global fitting analysis allowed construction of a quantitative model to support the earlier qualitative analysis by involving the intermediate and the final emissive STE state. There are two possible dynamic natures of the intermediate state: (1) the intermediate state directly decays to the ground state without involving the final emissive STE state and (2) the intermediate state relaxes to both the ground state and final emissive STE state. In the first scenario, the intermediate and final emissive STE states are formed simultaneously with a population ratio close to 50/50 based on the two PL decay components which show similar amplitudes from global analysis (Figure 2d). Then, they decay to the ground state independently. Considering the short lifetime of the intermediate state (∼180 ps), its radiative efficiency would be rather low. The high PLQY (∼50%) of the system would come from the final emissive STE state, which means that it must decay radiatively with 100% efficiency. This is unlikely to occur due to its long lifetime (∼15 μs) and other nonradiative processes. Therefore, this model is not taken into consideration for our system. The second scenario would be more reasonable following the relaxation model below (Figure 4c): The photogenerated excitons quickly form the intermediate STE state (STE_1_) with a time scale around 400 fs. Then, the intermediate STE_1_ state (with time scale of 180 ps) relaxes to the ground state via the fast radiative process or transfers to a long-lived emissive STE (STE_2_) with a lifetime at the microsecond time scale. This explanation accounts well for the highly efficient STE emission in Cs_2_ZrCl_6_ NCs.

Here, we discuss the origin of the two STEs. Based on several theoretical works,^8,42,44,49^ the density of state (DOS) of Cs_2_ZrCl_6_ reveals that the valence band maximum (VBM) is composed of Cl 3p orbitals, and the conduction band minimum (CBM) is dominated by hybridized antibonding orbitals of Cl 3p and Zr 4d. It indicates that the electronic transition within the [ZrCl_6_]^2–^ octahedra is a response for the light absorption and PL processes in Cs_2_ZrCl_6_. Therefore, the STEs in Cs_2_ZrCl_6_ NCs most likely originated from the distortion of [ZrCl_6_]^2–^ octahedra. Under the photoexcitation, the soft [ZrCl_6_]^2–^ octahedra are distorted to induce a strong exciton–phonon interaction, directly leading to the formation of STEs. The formation of STE_1_ and STE_2_ could be caused by the inhomogeneity of [ZrCl_6_]^2–^ octahedra.^50,51^ In other words, the fast time scale corresponds to a local distortion (STE_1_), while the longer 180 ps time scale corresponds to the reaching of the global minimum accessible for an exciton (STE_2_). A detailed excited state dynamic molecular simulation would gain a microscopic picture of the relevant distortion modes. But such a theoretical effort is out of the scope of the current experimental work.

In summary, we thoroughly investigated the intrinsic exciton dynamics of STE emission in 0D Cs_2_ZrCl_6_ NCs. The excitation energy is close to the optical bandgap to avoid hot excited state population. The excitation fluence is well maintained within the linear regime where the NC can contain only a maximum of one exciton. Based on these prerequisites, the hot carrier cooling process and Auger recombination are all ruled out. The exact formation and relaxation models of STEs are uncovered here: The ultrafast formation of STE happens at around 400 fs. After completing STE formation, the intermediate STE relaxes to the final emissive STE state at the time scale of 180 ps. Finally, the final emissive STE state emits photons via radiative recombination, which will last for more than one dozen of microseconds. Our work offers a precise dynamic picture of STEs in Cs_2_ZrCl_6_ NCs by involving an intermediate STE_1_ and final emissive STE_2_. The existence of multiple STEs can offer valuable guidance for understanding the underlying dynamics of STEs in their other perovskite counterparts. These findings further shed light on extending the potential applications of low dimensional perovskite-inspired materials showing STE-based broad PL emission.

## Supporting Information Available

Experimental details on nanocrystal synthesis, technical details of steady state spectroscopy, TCSPC, ICP-OES, TEM, Streak Camera, and TA experiments, UV–vis absorption and PLE spectra, PL intensity versus excitation fluences plotting, absorption cross section calculation of NCs (PDF)
Transparent Peer Review report available (PDF)
