# Supplementary material for: Nature of Self-Trapped Exciton Emission in Zero-Dimensional Cs2ZrCl6 Perovskite Nanocrystals
Source: J Phys Chem Lett. 2023 Aug 21;14(34):7665–71. doi: 10.1021/acs.jpclett.3c01878 (PMC10476180; doi:10.1021/acs.jpclett.3c01878)
Supplement: Supplementary file 1 — jz3c01878_si_001.pdf [file jz3c01878_si_001.pdf]

Supporting Information

Nature of Self-Trapped Excitonic Emission in Zero-Dimensional Cs_2ZrCl_6 Perovskite Nanocrystals

Yanmei He, [1,2] Siping Liu, [3,4] Zehan Yao, [1] Qian Zhao, [5] Pavel Chabera, [1] Kaibo Zheng, [1,5] Bin Yang, [3] Tönu Pullerits, [1,*] Junsheng Chen [1,2,*]

[1] Department of Chemical Physics and NanoLund, Lund University, P.O. Box 124, 22100 Lund, Sweden
[2] Nano-Science Center & Department of Chemistry, University of Copenhagen, Universitetsparken 5, 2100 Copenhagen, Denmark
[3] State Key Laboratory of Molecular Reaction Dynamics, Dalian Institute of Chemical Physics, Chinese Academy of Science, 116023 Dalian, P. R. China
[4] Guangxi Key Laboratory of Chemistry and Engineering of Forest Products, School of Chemistry and Chemical Engineering, Guangxi Minzu University, Nanning530006, P. R. China
[5] Department of Chemistry, Technical University of Denmark, DK-2800 Kongens Lyngby, Denmark

Corresponding authors

tonu.pullerits@chemphys.lu.se
junsheng.chen@chemphys.lu.se

Experimental section

Sample preparation

Firstly, 300 mg $Zr(CO_3)_2$(zirconium decarbonate, HWRK Chem, 99.90%), 10 ml ODE (1-octadecene, Alfa Aesar, 90%) and 325 μL CH_3COOH (acetic acid, Sigma, ≥ 99%) were added into a 3-neck round flask and heated at 105 °C to get a transparent solution. After around 30 minutes, 136 mg CsOAc (cesium acetate, Acros Organics, ≥ 98.5%), 2.8 mL oleic acid and 615 μL OLA (oleylamine, Acros Organics, ≥ 96%) were sequentially added. The mixture was degassed at low pressure condition to remove water and oxygen for 1 h at 105 °C. Later, the temperature was raised up to 200 °C under N_2 atmosphere. Once reaching 200 °C, 400 μL TMSCl (trimethylchlorosilane, > 98.0%, TCI) was swiftly injected within 20 seconds. Afterwards, the ice-water bath was used to cool it down. To get rid of the left reagents in solution, the raw mixture was isolated by centrifugating at 6500 rpm for 10 min. The white sediment was dispersed in 10 mL chloroform-d ($CHCl_3$) or heptane, and then centrifuged at 6500 rpm for 10 min again. Finally, the colloidal NCs could be obtained from the supernatant. For femtosecond transient absorption (fs-TA) and streak camera measurements, the prepared Cs_2ZrCl_6 NCs were transfer to heptane by evaporating $CHCl_3$ and dispersed in heptane with the same volume to exclude the potential solvent effect under high excitation photon energy.

Photophysical characterizations

Steady state absorption was measured by UV–vis absorption spectrophotometer (PerkinElmer, Lambda 1050). The photoluminescence (PL) spectra and photoluminescence quantum yield (PLQY) were measured by standard spectrometer (Horiba, Spex 1681) with excitation at 250 nm and estimated by relative calculation methods by using 2,4,6-Triphenylpyrylium tetrafluoroborate (fluorescence quantum yield = 58%) as reference.

The morphology and shape of NCs was obtained from transmission electron microscopy (Tecnai G2 T20 TEM). The NCs' concentration was measured by Inductively Coupled Plasma Optical Emission Spectroscopy (ICP-OES, Perkin Elmer Optima 8300). Powder X-ray diffraction (XRD) measurements were performed on a Rigaku D-MAX 2500/PC diffractometer equipped with Cu Kα radiation.

Time-resolved PL spectra within microseconds was measured using a FLS1000 Edinburgh Instruments spectrofluorometer. The Streak camera measurements were performed with the excitation pulses generated by a Ti:Sapphire laser (Spectra-Physics, Tsunami) with 60 fs pulse at 800 nm and repetition rate of 80 MHz. Time-resolved PL spectra at nanosecond scale were imaged onto the input slit of the streak camera (Hamamatsu C6860) set at 150 μm with the excitation of 266 nm.

Femtosecond transient absorption (fs-TA) spectroscopy

TA measurements were conducted by using a femtosecond pump-probe homemade set-up. Laser pulses (8 W, 796 nm, 60 fs, 4 kHz) come out of Solstice (Spectra Physics) amplifier seeded by a femtosecond oscillator (Mai Tai SP, Spectra Physics). The pulsed

laser light was divided into two parts to use for pump and probe beams. For the probe, the super-continuum white light is generated from a thin CaF_2 plate, covering the whole visible light region (430 ~ 775 nm). For pump pulse (30 mW, 266 nm, 130 fs) was produced by collinear optical parametric amplifier (Tripler 100M (X), Spectra Physics). Inside, the laser pulse light goes though BBO crystal to achieve double frequency, and then mixes with Mai Tai laser fundamental to get sum frequency. The output pump lights consist of 266, 400 and 800 nm, then the pure pump light of 266 nm is obtained by using two higly selective (for 266 nm) reflective mirrors. The global analysis (GLA) was performed by Glotaran software package (http://glotaran.org). The simple sequential or parallel decay model with various components was used to do SVD global fitting.[1]

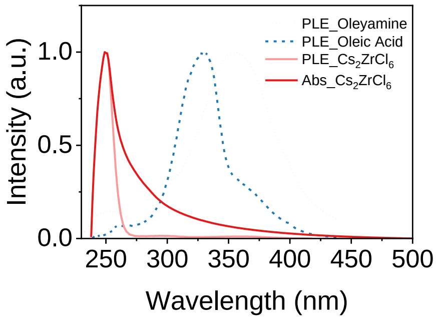

Figure S1. UV-vis absorption and PLE spectra of Cs_2ZrCl_6 NCs and organic reagents for comparison.

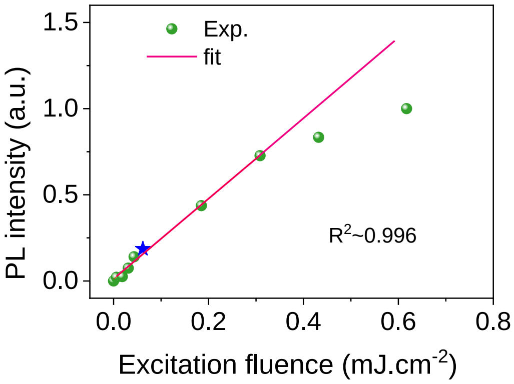

Figure S2. The plot of PL intensity versus excitation fluence pumped on Cs_2ZrCl_6 NCs. Green scatter and pink solid line stand for experimental data and linear fitting results, respectively. The blue star stands for the excitation fluence used in fs-TA measurements.

Absorption cross section calculation

The average photoexcited exciton number per NC is important to understand the STEs dynamics. It can be calculated through below equation:[2-3]

$$< N >= \sigma \times I \qquad (S1)$$

Where σ is absorption cross section of Cs_2ZrCl_6 NCs, I is the excitation fluence. The absorption cross section is estimated using Beer-Lambert law by combining with TEM, ICP-OES, and measured absorption spectrum.[4-6] The following equations are shown to explain this method.

$$A = \varepsilon CL \qquad (S2)$$

$$\sigma = \varepsilon \frac{2303}{N_A} \qquad (S3)$$

Where A, ε, C, L and N_A are absorbance of NCs dispersed in heptane, molar absorption coefficient, molar concentration, optical pathlength and Avogadro's number, respectively. The absorbance (A~0.93) is measured by steady state absorption spectroscopy. The molar concentration of NCs is estimated based on ICP-OES and TEM results. The value of obtained C is 8.5×10^{-3} mol.L^{-1}. Optical path is 1 mm. The obtained ε is around 1.1×10^{3} cm^{-1}.mol^{-1}.L calculated by equation S2. According to the equation S3, σ is around 4.2×10^{-15} cm^{2}. Finally, <N> is estimated to be 0.3 under the excitation fluence of 8.3×10^{13} photon/cm^{2}/pulse.

References

(1) Snellenburg, J. J.; Laptenok, S.; Seger, R.; Mullen, K. M.; van Stokkum, I. H. M., Glotaran: A Java-Based Graphical User Interface for the R Package TIMP. *J. Stat. Softw.* **2012,** *49* (3), 1 - 22.

(2) Chen, J.; Messing, M. E.; Zheng, K.; Pullerits, T., Cation-Dependent Hot Carrier Cooling in Halide Perovskite Nanocrystals. *J. Am. Chem. Soc.* **2019,** *141* (8), 3532-3540.

(3) Lenngren, N.; Garting, T.; Zheng, K.; Abdellah, M.; Lascoux, N.; Ma, F.; Yartsev, A.; Žídek, K.; Pullerits, T., Multiexciton Absorption Cross Sections of CdSe Quantum Dots Determined by Ultrafast Spectroscopy. *J. Phys. Chem. Lett.* **2013,** *4* (19), 3330-3336.

(4) Puthenpurayil, J.; Cheng, O. H.; Qiao, T.; Rossi, D.; Son, D. H., On the Determination of Absorption Cross Section of Colloidal Lead Halide Perovskite Quantum Dots. *J. Chem. Phys.* **2019,** *151* (15), 154706.

(5) Yeltik, A.; Delikanli, S.; Olutas, M.; Kelestemur, Y.; Guzelturk, B.; Demir, H. V., Experimental Determination of the Absorption Cross-Section and Molar Extinction Coefficient of Colloidal CdSe Nanoplatelets. *J. Phys. Chem. C* **2015,** *119* (47), 26768-26775.

(6) Cademartiri, L.; Montanari, E.; Calestani, G.; Migliori, A.; Guagliardi, A.; Ozin, G. A., Size-dependent Extinction Coefficients of PbS Quantum Dots. *J. Am. Chem. Soc.* **2006,** *128* (31), 10337-10346.
