# Supplementary material for: Nature of Self-Trapped Exciton Emission in Zero-Dimensional Cs2ZrCl6 Perovskite Nanocrystals
Source: J Phys Chem Lett. 2023 Aug 21;14(34):7665–71. doi: 10.1021/acs.jpclett.3c01878 (PMC10476180; doi:10.1021/acs.jpclett.3c01878)
Supplement: Supplementary file 2 — jz3c01878_si_002.pdf [file jz3c01878_si_002.pdf]

jz-2023-01878n.R1

Name: Peer Review Information for "Nature of Self-Trapped Excitonic Emission in Zero-Dimensional Cs_2ZrCl_6 Perovskite Nanocrystals"

First Round of Reviewer Comments

Reviewer: 1

Comments to the Author

The manuscript entitled “Nature of Self-Trapped Excitonic Emission in Zero-Dimensional Cs2ZrCl6 Perovskite Nanocrystals” presents the emission mechanism, materials design of self-trapped excitons materials. In my opinion, this work can be published in the The Journal of Physical Chemistry Letters after major revision. Here are some specific comments:

(1) The PL life time in Fig. 1d is long (15us), which is not accordance with the lifetime if Fig. 2c (ns scale).

(2) The STE1 and STE2 all come from the distortion of [ZrCl6] or other distortion mode, the author are suggested to discuss the origin of these two STEs.

(3) Will the excitation wavelength influence the carrier dynamic of self-trapped excitons.

Reviewer: 2

Comments to the Author

This work by He et al. reports the excited state dynamics in 0D Cs2ZrCl6 nanocrystals and provides a comprehensive picture of the STE emission dynamics. The major advance reported herein is the precise clarification of the dynamics of STE emission in this family of materials, which has not been earlier identified. The significance of this discovery is that it provides a tool to develop new potential applications for low dimensional metal halides.

This makes the work of interest for the community of halide perovskite researchers and beyond. This is an excellent study comprising a solid set of experiments that well support the claims. The text is well-written and comprises appropriate and easy-to-read figures. I recommend the publication of this manuscript in J. Phys. Chem. Lett after minor revisions addressing the following points:

- Sometimes in the text the word “halide perovskite” is used improperly and the term “perovskite-inspired material” would have been appropriate (see for example https://iopscience.iop.org/article/10.1088/1361-6528/abcf6d). The material investigated here, Cs2ZrCl6,

should be referred to as vacancy-ordered double perovskite. The authors are invited to check the terms throughout the text and modify accordingly.

- In the Introduction, more effort should be devoted to describing the motivation for studying this particular material, Cs_2ZrCl_6, and what is the importance of this material for the community.

- The findings of this work could possibly help understanding the STE in other low-dimensional perovskites. Can the authors elaborate this point a bit further in the Conclusions?

Author's Response to Peer Review Comments:

1. Supporting Information Statement: A brief, nonsentence description of the actual contents of each supporting information file is required. This description should be labeled Supporting Information and should appear before the Acknowledgement and Reference sections.

Response: Amended.

2. References: In both the main file and the supporting information, fix the style of all references to use JPCL formatting (check all references carefully).

Response: Amended.

3. Supporting Information: Please number SI pages in the following format: “S1, S2…”

Response: Amended.

Reviewer: 1

Recommendation: This paper is probably publishable, but major revision is needed; I do not need to see future revisions.

Response: We thank the reviewer for the positive feedback on our manuscript and we have modified the manuscript, based on the provided comments.

1. The PL lifetime in Fig. 1d is long (15us), which is not accordance with the lifetime in Fig. 2c (ns scale).

Response: The two experiments (Fig. 1d and Fig 2c) were done with two different techniques to cover different timescales of the self-trapped excitons' decay processes. In Fig. 1d, the PL decay was detected by time-correlated single-photon counting (TCSPC) method, which takes care of the radiative decay process at the timescale of μs. In Fig. 2c, the PL decay was collected by streak camera, which detects the decay process at tens of ps to few ns. The two methods are complementary to each other, and that is why there are different timescales in the two figures. We have added relevant information in paragraph 2 on page 5 of the revised manuscript.

2. The STE1 and STE2 all come from the distortion of [ZrCl6] or other distortion mode, the author are suggested to discuss the origin of these two STEs.

Response: Thanks for the good suggestions. We admit that the data in this work cannot uniquely determine the origin of these two STEs. We agree with the referee that the STEs originate from the distortion of $[ZrCl_6]^{2-}$ octahedra. Based on the calculated density of state (DOS) of Cs_2ZrCl_6 [reported in Refs. (8), (41, new), (43, new) and (48, new)]: the conduction band minimal (CBM) is dominated by hybridized antibonding orbitals of Cl 3p and Zr 4d, while the valence band maximum (VBM) is composed of Cl 3p orbitals. This indicates the electronic transition within the $[ZrCl_6]^{2-}$ octahedra is response for the light absorption and PL processes. Therefore, the STEs in Cs_2ZrCl_6 NCs are most likely originated from the distortion of $[ZrCl_6]^{2-}$ octahedra. The phonon frequency of Cs_2ZrCl_6 is around 49.4 meV [New Ref. (41)] and the estimated vibrational period is around 1.3 ps. In this case, the STEs could form within 400 fs. For the origin of two different STEs, it might be explained by the inhomogeneity of the $[ZrCl_6]^{2-}$ octahedra in NCs [New Ref. (49), (50)]. In other words, the fast timescale corresponds to a local distortion (STE_1), while the longer 180 ps timescale corresponds to the reaching of the global minimum accessible for an exciton (STE_2). A detailed excited-state dynamic molecular simulation would gain a microscopic picture of the relevant distortion modes. However, such theoretical effort is out of the scope of current experimental work. We added the relevant discussion in the last paragraph on Page 9-10.

3. Will the excitation wavelength influence the carrier dynamic of self-trapped excitons.

Response: In our previous work [Ref. (8)], we measured the excited state dynamics of STEs in Cs_2ZrCl_6 NCs by using the excitation wavelength of 250 nm. With higher excitation energy, we could observe the cooling process (~300 fs) of hot charge carriers by observing the build-up

process of the excited-state absorption signal. For the decay process, we used the Global Analysis method to fit the data and we can observe similar STE dynamics: the STEs forms within 400 fs; The STE_1 relaxes to STE_2 with the timescale of ~250 ps; Then, a slow decay process with timescale >>10ns. Due to the limited signal-to-noise ratio in the data, the fitted timescale of STE_1 is a bit longer than that obtained under the band-edge excitation. The 250 ps timescale can be fitted to a value of 180 ps as well. When we used band-edge excitation (266 nm), there was no extra population of hot excited states. It is confirmed by the sharp rising onset of ESA signal and spectral-red-shift of 440, 540 and 660 nm. We also tried to use 300 nm laser with high excitation intensity to measure the carrier dynamics (Figure R2), but we did not observe any signals. Because the 300 nm pump light has lower energy than the optical band gap of Cs_2ZrCl_6 nanocrystals (E_g~4.70 eV, 270 nm).

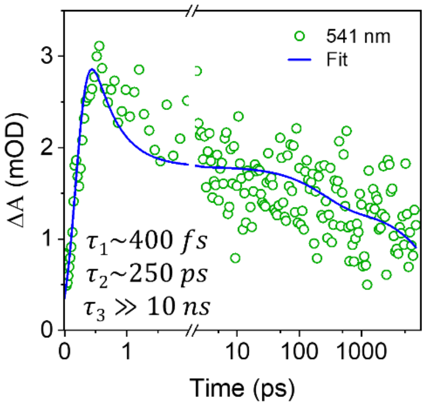

Figure R1. Temporal kinetics of Cs_2ZrCl_6 NCs (excited at 250 nm) probed at 540 nm with fitting results.

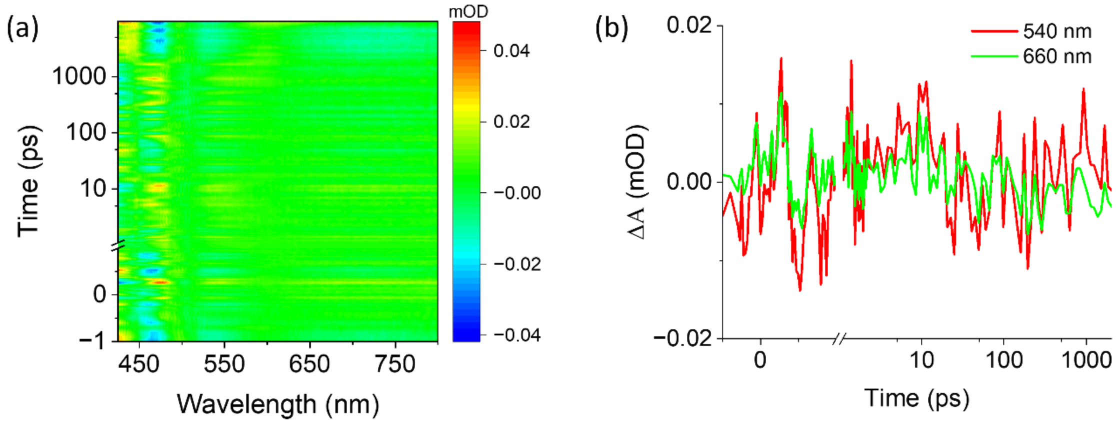

Figure R2. (a) Pseudocolor representation of fs-TA spectra of Cs_2ZrCl_6 NCs excited at 300 nm with the excitation intensity of 4.4×10^{14} photon/cm^2/pulse. (b) Signal at at 540 and 660 nm.

Reviewer 2:

Recommendation: This paper is publishable subject to minor revisions noted. Further review is not needed.

Response: We thank the reviewer for the positive feedback on our manuscript and for acknowledging important aspects of our study.

1. Sometimes in the text the word "halide perovskite" is used improperly and the term "perovskite-inspired material" would have been appropriate (see for example https://iopscience.iop.org/article/10.1088/1361-6528/abcf6d). The material investigated here, Cs2ZrCl6, should be referred to as vacancy-ordered double perovskite. The authors are invited to check the terms throughout the text and modify accordingly.

Response: Thanks for suggestions. We carefully checked and corrected the terms throughout the whole paper. We also cited the publication as Ref. 11.

2. In the Introduction, more effort should be devoted to describing the motivation for studying this particular material, Cs2ZrCl6, and what is the importance of this material for the community.

Response: As reported in our previous work (Ref. (8)), 0D Cs_2ZrCl_6 NCs are stable and lead-free, showing typical and efficient STE emission among the low dimensional perovskite-inspired material. Cs_2ZrCl_6 has potential in many different applications such as LEDs and X-ray scintillator screens et al. It is significant to explore the exciton dynamics of Cs_2ZrCl_6 NCs. Our efforts on exploring the exciton dynamics in Cs_2ZrCl_6 NCs would also offer valuable guidance for perovskite counterparts showing STE emissions. The related descriptions of motivation are added in the last paragraph on Page 3.

3. The findings of this work could possibly help understanding the STE in other low-dimensional perovskites. Can the authors elaborate this point a bit further in the Conclusions?

Response: Thanks for your advice. We added the related elaborations.
